# Genicular Arteries Embolization for Patients with Osteoarthritis, Their Selection, and Follow-Up Based on MRI Findings

**DOI:** 10.3390/medicina61050941

**Published:** 2025-05-21

**Authors:** Aurelija Domarkienė, Lukas Kalytis, Gytis Kanapienis, Marius Kurminas, Algirdas Edvardas Tamošiūnas

**Affiliations:** 1 Department of Radiology, Nuclear Medicine and Medical Physics, Faculty of Medicine, Institute of Biomedical Sciences, Vilnius University, 01513 Vilnius, Lithuania; lukas.kalytis@mf.stud.vu.lt (L.K.); marius.kurminas@santa.lt (M.K.); algirdas.tamosiunas@mf.vu.lt (A.E.T.); 2 Center for Radiology and Nuclear Medicine, Vilnius University Hospital Santaros Klinikos, 08661 Vilnius, Lithuania

**Keywords:** Genicular artery embolization (GAE), knee osteoarthritis (OA), dynamic contrast enhanced MRI (DCE-MRI)

## Abstract

Osteoarthritis (OA) is a leading cause of disability worldwide, with its prevalence rising due to aging populations. Management ranges from conservative treatments such as weight management and pharmacologic therapy to surgical interventions such as total joint replacement. However, treating moderate knee OA remains challenging for patients unresponsive to conservative care but not yet surgical candidates. Genicular artery embolization (GAE) has emerged as a minimally invasive procedure targeting abnormal angiogenesis and inflammation in OA. This article explores GAE’s mechanism, patient-selection criteria, and effectiveness in pain reduction and functional improvement. Studies suggest that GAE has the potential to significantly improve pain and function in mild to moderate OA, with sustained benefits. Patient selection is crucial for optimal outcomes, with imaging playing a key role. While conventional MRI assesses structural damage, Dynamic Contrast-Enhanced MRI (DCE-MRI) offers superior insights by evaluating synovitis, quantifying cartilage degradation, and monitoring treatment response. Due to its strong correlation with pain scores and status as the best surrogate marker for inflammation in synovitis, DCE-MRI holds significant potential to enhance patient selection and treatment monitoring for GAE.

## 1. Introduction

Osteoarthritis (OA) is a widespread and leading cause of disability among adults globally [1]. With an aging population, the incidence, prevalence, and economic burden of OA treatment and related disabilities are expected to rise [2]. Identifying effective treatments across all OA stages, from mild to severe, is therefore essential.

Managing OA involves a range of treatment options aimed at symptom relief and functional improvement. Effective OA management emphasizes health education and lifestyle modifications, with regular moderate exercise and weight reduction to alleviate stress on articular cartilage [3]. Pain relievers such as acetaminophen and NSAIDs can support these core treatments by targeting pain pathways in mild to moderate OA. Additionally, local therapies such as intra-articular corticosteroid injections and hyaluronic acid injections (viscosupplementation) have proven to be effective alternatives for alleviating mild to moderate knee OA symptoms [4,5]. For severe, end-stage OA, total joint replacement surgery is often necessary.

Despite the wide spectrum of treatment methods, managing moderate OA that does not respond to nonsurgical treatments and is not severe enough for joint replacement remains challenging. Arthroscopic lavage and debridement have been used for years to treat this type of moderate knee osteoarthritis. However, a Cochrane meta-analysis, which included data from double-blinded, randomized, placebo-controlled trials, found no significant difference in effectiveness compared to a placebo (sham procedure) leading to the recommendation that these interventions should be excluded from osteoarthritis treatment protocols [6]. Therefore, there is a pressing need to develop new, effective, and safe treatments for patients with moderate knee osteoarthritis who are resistant to conservative treatment.

## 2. Understanding GAE and Its Mechanism of Action

Recent studies on the pathogenetic model of OA have shifted focus to low-level inflammation, which leads to joint remodeling. Chronic inflammation disrupts chondrocyte function, causing normal cell signaling to shift towards pro-inflammatory cytokines, thereby promoting angiogenesis [7]. These new blood vessels are often found in the synovium, infrapatellar fat pad, meniscus, joint capsule, and the periosteum adjacent to the condyle [8]. Multiple small animal models have shown that the extent of angiogenesis is linked to more severe OA [9,10]. These neovessels serve as pathways for persistent joint inflammation and neuronal migration. This process results in atypical cartilage innervation, heightening pain sensitivity. Factors such as hypoxia, inflammation, and mechanical stress within the joint further sensitize these nerves, exacerbating the pain response and worsening symptoms [11].

The recognition of abnormal angiogenesis and vascular invasion at the osteochondral junction as key features of OA pathophysiology has paved the way for innovative treatment, including genicular artery embolization (GAE) [7]. During the procedure, a thin catheter is inserted into the genicular arteries through a small incision in the groin. With the help of X-ray imaging, the catheter is guided to the affected artery, where tiny embolizing particles are injected until abnormal blood vessels show reduced filling and blush on imaging [8]. By targeting these newly formed blood vessels, GAE is hypothesized to reduce inflammation and pain in osteoarthritis patients, offering a novel approach to disease management.

The exact mechanisms through which transcatheter arterial embolization (TAE) relieves symptoms in patients are not fully understood. However, analyzing the pattern of pain and symptom relief might help clarify this treatment’s rationale. Two key phases of improvement have been observed. The first occurs almost immediately after the procedure, with patients experiencing reduced tenderness within minutes of receiving the embolization agent. A possible explanation for the immediate relief is that decreasing abnormal blood flow reduces sensory nerve stimulation, leading to a quick reduction in pain. The second phase happens weeks to months later. This delayed improvement observed over time may result from the inhibition of new blood vessel formation, which can reduce inflammation by limiting the influx of inflammatory cells into synovial tissue. This mechanism suggests that TAE may confer sustained anti-inflammatory and analgesic effects [8].

Continued follow-up using magnetic resonance imaging in GAE-treated patients showed a significant reduction in synovitis [12]. These findings indicate that embolization may not only alleviate pain but also potentially alter the course of the disease. As a result, patients undergoing GAE might be able to delay knee joint replacement compared to those who have not undergone the procedure [13]. However, confirming this potential benefit will require long-term follow-up studies for a clearer understanding.

## 3. GAE Efficiency and Risk

A meta-analysis by Torkian et al. [14] found substantial benefits in both pain levels and overall function after GAE. They measured these improvements using VAS (Visual Analog Scale) for pain and WOMAC (Western Ontario and McMaster Universities Osteoarthritis Index) for stiffness, pain and overall function. On the VAS pain scale, mean differences (MDs) showed improvements of 32 points in the first week, increasing to 58 points two years later after the procedure. This indicates pain reduction of 54% and 80% respectively. The overall WOMAC scores exhibited a comparable pattern of improvement- WOMAC scores had MDs between 28.4 and 36.8 points, suggesting 58% to 85% better pain management and physical capability. In addition, Genicular artery embolization (GAE) significantly reduced the need for various pain management treatments in patients with knee osteoarthritis (OA). Specifically, opioid use decreased by 27%, nonsteroidal anti-inflammatory drug use dropped by 65%, and intra-articular hyaluronic acid injections declined by 73%. These reductions were all statistically significant (*p* < 0.00001) [14]. These results suggest that GAE is associated with significant and sustained pain improvement with better functional status. However, this meta-analysis has significant limitations, as the included studies lacked a control group with alternative treatment modalities. Consequently, these studies did not account for the placebo effect, which is known to play a role in reducing pain in patients with knee OA [15]. Randomized controlled trials (RCTs) are needed to better evaluate the efficacy of GAE, yet existing studies present ambiguous results. A notable RCT conducted by van Zadelhoff et al. [16], which used a sham procedure as the control, found no significant difference in pain reduction between the control and GAE groups, likely due to a substantial placebo effect. However, this study had several limitations. First, pain outcomes were assessed only at baseline, 1 month, and 4 months, while previous studies suggest that GAE outcomes tend to improve over time [14]. It is possible that after a longer follow-up period, the placebo effect diminishes while the effects of GAE persist. Second, patient selection relied solely on radiographic Kellgren–Lawrence (KL) grading, without MRI evaluation, meaning that synovitis and other soft tissue changes were not assessed in detail. Third, the majority of patients had KL grade 3 osteoarthritis, which is known to respond less favorably to GAE compared to earlier stages (KL 1–2) [12]. Finally, the study had a relatively small sample size (58 patients), limiting its statistical power. Another RCT by Bagla et al. [17] demonstrated that in patients with mild to moderate knee osteoarthritis, GAE resulted in significantly greater symptomatic improvement compared to the sham procedure. The GAE group experienced a 50.1 mm greater reduction in pain (VAS score) and a 24.7-point greater improvement in function (WOMAC score) after 1 month, underscoring its potential in pain and disability reduction. This study also included a 12-month follow-up period, providing longer-term data. However, limitations included a small sample size (21 patients) and the absence of MRI-based patient selection, which could have improved the precision of inclusion criteria. Overall, these findings suggest that GAE holds promise as a treatment for mild to moderate knee OA, but further research is needed. Future studies should include larger sample sizes, MRI-based patient selection, and long-term follow-up to better assess the efficacy and durability of GAE in managing osteoarthritis-related pain.

As an invasive procedure, GAE carries certain risks and potential complications. According to data from the previously mentioned meta-analysis by Torkian et al. [14], 54 out of 214 participants experienced minor adverse events, resulting in an overall complication rate of 25.2%. The most common issue was self-resolving transient cutaneous ischemia, while other reported minor complications included puncture site hematomas, skin redness, and transient fever in a few cases. Importantly, no severe adverse events, such as weakness or joint instability, were reported following GAE. Furthermore, although GAE intentionally reduces blood flow to specific areas of the knee for pain relief, no cases of avascular necrosis (AVN) were reported in the studies analyzed by this meta-analysis [14]. However, several studies have documented isolated cases of transient osteonecrosis following the procedure, all of which resolved within a few months without lasting ischemic complications [18,19]. Considering these findings, GAE can generally be regarded as a safe treatment option [14].

From a health economics perspective, surgical procedures represent the primary driver of high overall costs compared to routine medication use in patients with knee OA-related pain. However, limited data exist on the cost-effectiveness of GAE, particularly in terms of quality-adjusted life years (QALYs). In a study by Davies and Isaacson [20], GAE was found to be more expensive than NSAIDs but less costly than cyclooxygenase-2 (COX-2) selective inhibitors when factoring in expected future complication costs. However, to establish a more comprehensive economic comparison, future studies should incorporate QALY assessments, long-term follow-up, and a broader evaluation of product pricing across various treatment modalities.

## 4. Role of Imaging and Most Important MRI Parameters

Radiography is the most prevalent primary technique used for diagnosing OA [21]. Radiographic assessment of OA primarily focuses on bone changes, while cartilage health is indirectly evaluated through the measurement of joint space width [22]. The narrowing of this joint space is often used as an indicator of OA progression. However, recent studies using MRI have revealed that joint space narrowing is not solely attributable to cartilage loss. Instead, it results from a combination of factors, including meniscal damage, meniscal extrusion, and cartilage deterioration [23]. Therefore, MRI imaging is essential for a comprehensive evaluation of the intricate structures within the knee joint. Several different sequences are used to evaluate OA. Conventional MRI evaluates knee OA using T1-weighted (T1), T2-weighted (T2), and proton density-weighted (PD) imaging. The European Society of Skeletal Radiology (ESSR) guidelines recommend obtaining fat-saturated proton density-weighted (PD FS) images in three planes (axial, sagittal, and coronal) [24]. The key components examined on MRI are cartilage lesions, bone marrow lesions, subchondral bone changes and osteophytes. Additionally, meniscal lesions, effusion synovitis, and changes in other anatomical structures are noted [25].

In the setting of knee OA, MRI is usually performed without the use of intravenous contrast even though it is generally accepted that synovitis is ideally assessed with contrast-enhanced (CE)-MRI, as only this enables the clear differentiation of synovitis from joint effusion [26]. In addition, when intravenous contrast is used, it provides the possibility to add a dynamic contrast-enhanced sequence to the MRI protocol. Dynamic contrast-enhanced MRI (DCE-MRI) is an advanced imaging technique used to assess tissue perfusion, vascular permeability, and blood flow by tracking the movement of a contrast agent over time. A meta-analysis by Shakoor et al. [27] assessed the correlation between synovitis detected on non-CE MRI, CE-MRI, and DCE-MRI with the gold standard of histologic assessment. Across eight studies comparing CE-MRI to histology, a moderate positive correlation was observed for both macroscopic (r = 0.53) and microscopic (r = 0.56) assessments. In contrast, the pooled correlation coefficient between non-CE MRI and histology was lower (r = 0.44), suggesting limited accuracy. Notably, DCE-MRI demonstrated the strongest correlation with histology (r = 0.71) based on two studies, indicating superior accuracy in synovitis assessment. These findings suggest that DCE-MRI may provide the most reliable imaging evaluation of synovitis in knee OA.

Another critical aspect to consider is the selection of DCE-MRI parameters that provide the most accurate evaluation of knee OA. In this context, Mackay et al. [28] conducted a study to assess the diagnostic performance of DCE-MRI biomarkers in patients with knee OA. The study included 14 patients with knee OA and six healthy volunteers, all of whom underwent DCE-MRI at baseline, 1 month, and 6 months. Synovial segmentation was performed using a semi-automatic method to ensure consistency in analysis. DCE-MRI was obtained using a pharmacokinetic modeling approach with standard biomarkers such as *K*^trans^ (units min^−1^), the volume transfer constant for contrast agent between blood plasma and extravascular extracellular space; *v_p_*, fractional volume of blood plasma; *v_e_*, the fractional volume of extravascular extracellular space; and IAUC_60_ (mM.s), the initial area under the contrast agent concentration–time curve 60 s post-contrast agent arrival in the tissue extracted during image analysis. The findings identified *K*^trans^ as the most reliable DCE-MRI biomarker, demonstrating superior test-retest reproducibility, discriminative ability, and sensitivity to change. The authors emphasized that biomarkers assessing synovitis severity, such as K^trans^, provide greater diagnostic and prognostic value than those measuring only synovitis extent, such as volume. These results suggest that pharmacokinetic analysis using K^trans^ should be prioritized in future clinical trials evaluating anti-inflammatory treatments for OA, as it may offer a more precise assessment of treatment response and disease progression.

## 5. Patient Selection for GAE

Overall, most studies focus on patients with mild to moderate knee osteoarthritis, typically classified as KL grade 1–3 on X-ray, who have experienced knee pain for at least six months. These patients generally have not responded to conservative treatments such as physiotherapy, analgesics, weight loss, or intra-articular injections, and are therefore considered for further evaluation using MRI and potential treatment with the GAE procedure [12,14].

GAE tends to be more effective in reducing pain and improving function in patients with mild to moderate OA. Severe OA often involves significant joint damage that may not respond as well to this procedure. A study by Lee et al. [29] showed that patients with mild-to-moderate OA (KL grade 1–3) experienced effective pain relief through GAE. However, for those with severe OA (KL grade 4), the treatment’s effects were short-lived. While pain initially decreased for the first month, it gradually returned to pre-treatment levels over the 3 months. Another study by Okuno et al. [12] showed similar results: patients with more severe degenerative changes (KL grade 3) experienced less frequent clinical success 6 months after the procedure. In contrast, patients with milder osteoarthritic changes (KL grades 1 or 2) had a higher rate of clinical success at the 6-month mark. The results of these studies suggest that the severe loss of articular cartilage in advanced OA resulting in direct bone-to-bone contact could be the cause of this relapse of pain and because of that the treatment may be more effective for patients in earlier stages of OA compared to those with more advanced joint degeneration.

Another study conducted by Zadelhoff et al. [30] tried to find prognostic MRI features, which predict the effectiveness of pain relief before the GAE procedure in patients with OA. According to this study the cartilage full-thickness defects whole joint score was found to be the greatest predictor for less pain reduction, with the highest full-thickness cartilage defect causing the least amount of pain reduction post treatment. The presence of effusion synovitis on MRI at baseline was found to be related to lesser pain reduction. There was a small positive but insignificant correlation between Hoffa synovitis and reduction of pain which means that patients particularly with synovitis are not more responsive to GAE treatment. Larger amounts of osteophytes, bone marrow lesions (BMLs), and subregional cartilage lesions as seen on MR imaging all correlate with lesser pain relief after embolization. All in all, these results highlight the importance of early intervention in OA and suggest that the stage of the disease should be a key consideration when selecting the most appropriate patients for GAE.

Assessing knee characteristics on MRI before the GAE procedure is crucial to ensure that patients selected for GAE will benefit from the procedure while minimizing risks. Specific MRI criteria help identify patients whose OA symptoms are driven by inflammation and vascular abnormalities rather than structural damage that may require alternative treatments. For example, active synovitis indicates ongoing inflammation that GAE can effectively target, while grade IV chondromalacia, large meniscal tears, or subchondral bone lesions indicate significant joint instability, making the procedure less beneficial. Additionally, ruling out conditions such as avascular necrosis, acute trauma, or rheumatological diseases prevents inappropriate treatment and directs patients to more suitable management options [12,31,32,33].

There were no studies found specifically addressing the difference in outcomes between treating one knee versus both knees in a single patient. However, some studies—such as the one by Okuno et al. [12]—performed radiological assessments and GAE procedures on both knees of the same patient, treating each knee as a separate unit or participant.

**Figure 1:**
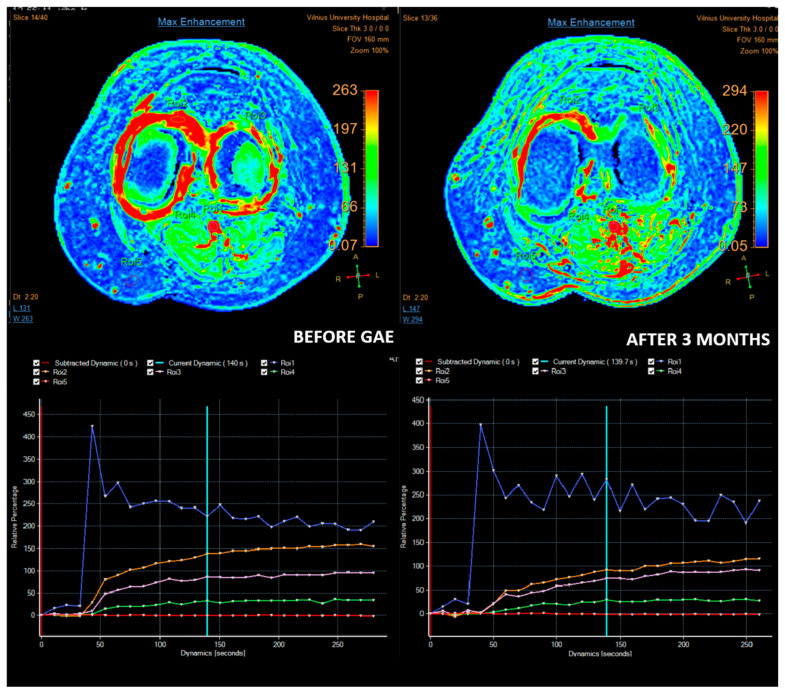
Images of a patient with moderate (grade II–III chondromalacia) OA treated with GAE. Synovial perfusion before GAE and 3 months after GAE. On maximum enhancement maps (top row), before GAE, high enhancement is observed in the synovium, especially on the medial side (ROI 2). After three months, reduced enhancement in the synovium is observed on the maximum enhancement map, especially medially. Relative percentage enhancement curves (bottom row) also show reduced enhancement on both sides (medial ROI2 and lateral ROI 3), with a significantly lower medial-enhancement curve.

**Figure 2:**
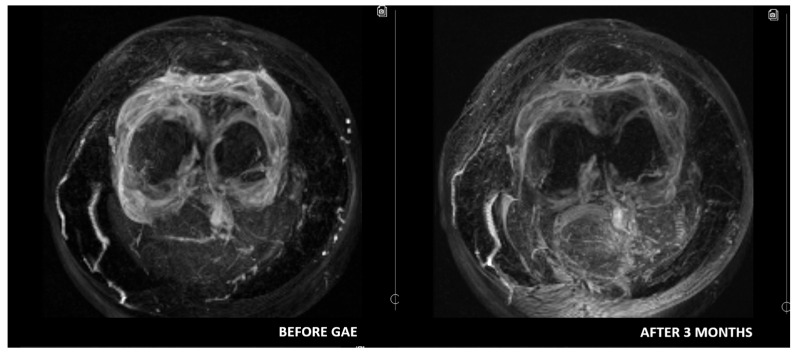
T1 vibe subtraction MIP images. A diffuse avid enhancement in the synovium is observed before GAE. The enhancement is reduced after 3 months following GAE.

**Figure 3:**
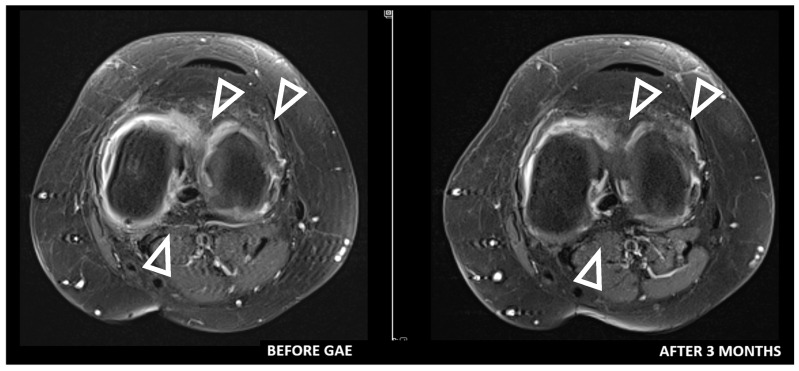
Contrast-enhanced T1 fat-saturation images. Reduced synovial thickness and enhancement (enhancing, arrowheads) are observed after 3 months following GAE (arrowheads).

Some authors suggest that DCE-MRI may provide valuable additional insights for diagnosing and selecting patients with OA. Compared to conventional MRI, DCE-MRI offers significant advantages by assessing synovitis activity, and quantifying cartilage degradation. Moreover, it enables monitoring of treatment response by capturing dynamic changes in blood flow [34,35]. Riis et al. [36] evaluated synovitis using DCE-MRI and found statistically significant correlations between most DCE-MRI variables and KOOS-Pain scores. Similarly, static MRI variables also demonstrated significant correlations with KOOS-Pain scores. Given that DCE-MRI variables reflect perfusion and can most accurately serve as surrogate markers of inflammation, it is reasonable to hypothesize that DCE-MRI may be the most accurate radiological method for patient selection and treatment monitoring (Figure 1, Figure 2 and Figure 3). However, to date, no studies have investigated the use of DCE-MRI in patients undergoing GAE. This gap in literature may be attributed to the relative novelty of both techniques, with current research prioritizing clinical outcomes over radiological assessment. Further research is needed to test this hypothesis and establish standardized MRI-based criteria for optimizing patient selection and monitoring in GAE treatment.

## 6. Conclusions

GAE represents an innovative and minimally invasive treatment for knee OA, particularly for patients who do not respond to conservative therapies but are not yet candidates for joint replacement. By targeting pathological angiogenesis and inflammation, GAE has the potential to demonstrate significant and sustained improvements in pain relief and function. Patient selection is critical, as those with mild to moderate OA respond more favorably than those with severe joint degeneration. Imaging plays a crucial role in identifying suitable candidates for GAE. While conventional MRI focuses on detecting structural abnormalities, DCE-MRI offers a more detailed assessment by more accurately identifying synovitis, measuring cartilage degeneration, and tracking treatment effectiveness. With its strong correlation to pain scores and its role as the most reliable surrogate marker of inflammation in synovitis, DCE-MRI has the potential to refine patient selection and improve the monitoring of GAE outcomes. However, further research is needed to identify the most predictive DCE-MRI variables for assessing patient outcomes after GAE. A deeper understanding of GAE’s mechanisms, along with refined patient selection strategies, will be essential for optimizing its clinical application and integrating it into standard OA management.
